# Saunders’s Question Compends

**Published:** 1902-04

**Authors:** 


SAUNDERS'S QUESTION COMPENDS. Essentials of Physiology. Prepared especially for Students of Medicine, and arranged with questions following each chapter. By SIDNEY P. BUDGETT, M. D., Professor of Physiology, Medical Department of Washington University, St. Louis. 16mo volume of 233 pages, illustrated with many full-page half-tones. Philadelphia and London: W. B. Saunders & Co. 1901. [Price, cloth, $1.00 net.]

An entirely new work has been added to Saunders's series of question compends in this little volume on physiology. The book has one very great superiority over many similar works—the questions are placed at the end of each chapter instead of being incorporated in the text. M. J. F.

